# False smut of rice: integrating molecular pathogenicity and epidemiology for next-generation disease management

**DOI:** 10.3389/ffunb.2026.1875221

**Published:** 2026-07-01

**Authors:** K. Nishmitha, Sudeep Adhikari, Pankaj Kumar Mishra, Suryakant Manik, Hem Chandra Lal, Lham Dorjee

**Affiliations:** 1 Rice Breeding Platform, South Asia Regional Center, International Rice Research Institute, Varanasi, India; 2 Plant Breeding Graduate Program, University of Florida, Gainesville, FL, United States; 3 Faculty of Agriculture, Ramakrishna Vivekananda Educational and Research Institute, Ranchi, India; 4 Indian Council of Agricultural Research-Indian Institute of Agricultural Biotechnology, Ranchi, India; 5 Department of Plant Pathology, Birsa Agricultural University, Ranchi, India

**Keywords:** effector, genome editing, infection biology, rice, *Ustilaginoidea virens*

## Abstract

False smut of rice, caused by *Ustilaginoidea virens*, has emerged as a serious threat to global rice production, resulting in substantial yield and grain quality losses. This review summarizes recent advances in understanding the pathogen’s life cycle, infection biology, and epidemiological factors, while highlighting the limitations of current management strategies that rely predominantly on fungicide applications. *U. virens* exhibits a unique biotrophic infection strategy, colonizing floral tissues without specialized infection structures and manipulating host physiology through a diverse repertoire of effectors that suppress immunity, alter hormonal signaling, and hijack sugar transport systems to facilitate nutrient diversion and smut ball formation. Integrating molecular mechanisms with epidemiological factors such as climate variability and inoculum dynamics provides important insights into disease development and spread. Recent progress in artificial intelligence has enabled the development of predictive modeling frameworks, while advances in spectral imaging and molecular diagnostics offer promising tools for early detection and disease forecasting. In the absence of identified resistance rice cultivars, genome editing and microbiome-based strategies are viable alternative approaches for durable disease control. These advances together provide a basis for a transition to an integrated management framework incorporating forecasting models, genome editing, and microbiome-informed interventions for more sustainable and effective control of rice false smut.

## 1 Introduction

False smut of rice, caused by *Ustilaginoidea virens* (teleomorph: *Villosiclava virens*), was once regarded as a minor disease due to its sporadic occurrence. However, in the past decade, it has emerged as a major threat, with reported yield losses of up to 49% (Sun et al., 2020). In India, disease incidence has increased significantly, ranging from 5% to 85%, with yield losses of 0.2-49% (Ladhalakshmi et al., 2012). This rise in prevalence has been due to the widespread cultivation of fertilizer-responsive varieties with a narrow genetic base, intensive agricultural practices, and changing climatic conditions (Chaudhari et al., 2021). Beyond yield reduction, false smut poses serious concerns for grain quality and food safety due to mycotoxin contamination, which can adversely affect both human and livestock health. The disease initiates during the booting stage, and symptoms become visible only after panicle emergence, making early detection and timely management challenging.

Current management strategies rely largely on chemical control. Fungicide combinations such as trifloxystrobin + tebuconazole and difenoconazole + propiconazole have shown promising results when applied at the booting stage and at 50% panicle emergence (Sharma et al., 2024; Akphumkitanun et al., 2025). However, their effectiveness depends heavily on the precise time of application, leaving farmers with a narrow window for intervention. In addition, environmental conditions favorable for pathogen development, such as high humidity, rainfall, and moderate temperatures (25-28 °C), often coincide with the flowering stage, making disease outbreaks difficult to predict and manage. The lack of highly resistant rice varieties and the limited success of alternative approaches, including biological control agents, further constrain effective disease management. These challenges highlight critical gaps in our understanding of the disease, particularly the molecular mechanisms underlying pathogen infection and host interaction, as well as the epidemiological factors driving its increasing incidence and severity.

This review aims to summarizes recent advances in the molecular pathogenicity of *U. virens* and current knowledge on the epidemiological factors contributing to the emergence and severity of false smut disease. It also explores how these insights can be leveraged to develop improved strategies for disease prevention and control.

## 2 Disease cycle of *Ustilaginoidea virens*

The smut balls containing thick-walled chlamydospores overwinter along with rice seeds as contaminants and spread by air currents or water splash in the field. During the booting stage, they germinate and produce secondary conidia, which infect rice spikelets under favorable conditions such as high humidity, rainfall, and temperature around 28 °C, converting them into smut balls and impacting grain formation (Yong et al., 2018a; Sathiyaseelan et al., 2025). However, the factors that induce chlamydospore germination during the favorable booting stage are yet to be identified. The recent report suggests that the exopolysaccharides secreted by the rhizospheric Sphingomonadaceae bacteria facilitate the breakdown of dormancy and lead to germination. Interestingly, it was found that susceptible rice varieties recruit these bacteria in the rhizosphere (Pan et al., 2025). The pathogen employs a distinct strategy to infect the spikelet during the late booting stage. It initially grows epiphytically on the outer spikelet before entering the space between the lemma and palea (Tanaka et al., 2017). Microscopic studies reveal the pathogen preferentially infects the stamen first, followed by lodicules, anthers, stigma, and ovary. The pathogen’s inability to form smut balls in stamen-deficient rice mutants indicates the stamen’s critical role in the initiation and establishment of infection (Fan et al., 2020). This might be due to cells in the stamen filament being loosely arranged compared with the compact, thick-walled cells in the ovary and anther that are lined with microfibrils and cellulose (Rong et al., 2017). During infection, the pathogen grows intercellularly without forming appressoria or haustoria (Tang et al., 2013). Instead of killing host tissues, it induces host genes involved in nutrient efflux, supporting its growth while keeping floral organs alive (Fan et al., 2015). *UvPal1*, an endocytosis-related protein, appears to play an important role in smut ball formation. It interacts with the septin protein *UvCdc11* (cell division control protein 11), and deletion of either gene results in impaired fungal growth and failure to colonize the spikelet (Chen et al., 2020).

Infected spikelets are converted into smut balls that are 2–5 times larger than individual rice grains (Tang et al., 2013; Fan et al., 2020). The immature smut balls are initially white colored and filled with chlamydospores gradually, changing to yellow, yellowish-green, and black mature balls with sclerotia (Fan et al., 2015). The pathogen not only prevents grain filling but also increases spikelet sterility, contributing to significant grain loss (Dhua et al., 2015). The cool environment during later stages will result in the production of horseshoe-shaped sclerotia within the smut balls (Cheng, 2016). These sclerotia overwinter in the field and, under favorable humidity and temperature, germinate to produce perithecia filled with ascospores (Zhang et al., 2014). The chlamydospores and sclerotia serve as the primary source of inoculum, infecting the spikelet during the late booting stage and converting the immature spikelets into smut balls (Zhang et al., 2014; Yong et al., 2018a).

The conidia also infect rice coleoptiles and roots at the seedling stage, and the pathogen exhibits asymptotic epiphytic growth (Prakobsub and Ashizawa, 2017; Tanaka et al., 2017). The presence of pathogen growth on leaf and sheath without systemic vascular colonization has been reported by many researchers (Yong et al., 2018b). The pathogen grows on the plant epiphytically, thereby increasing the chance of infecting rice panicles during the booting stage (Tanaka et al., 2017). It is intriguing to understand how the pathogen grows epiphytically on the rice leaf sheath without triggering a defense response. The epiphytic infection on alternative weed hosts such as *Panicum tenellum*, *Digitaria marginate*, *Panicum trypheron*, *Imperata cylindrica*, and *Echinochloa crusgalli* has been recorded and may serve as a potential reservoir during off-seasons, although this has not yet been conclusively proven (Atia, 2004; Shetty and Shetty, 1985, Shetty and Shetty, 1987).

## 3 Mycotoxin production by *Ustilaginoidea virens*

In addition to yield loss, the pathogen also produces toxins as secondary metabolites that are detrimental to human and animal health, threatening food safety (Fengmin et al., 2024). Three toxins, ustiloxins, ustilaginoidins, and sorbicillinoids have been reviewed in *U. virens*. Their biosynthetic pathway and various regulators of toxin production have been elaborately identified previously (Li et al., 2019; Sun et al., 2020). However, their role in pathogenicity and the establishment of infection in rice are poorly elucidated. Ustiloxins are ribosomally synthesized 13-membered cyclic tetrapeptides (Tsukui et al., 2015), among which Ustiloxin A is predominantly present in the smut balls. The toxin inhibits microtubule formation and cytoskeleton assembly in eukaryotic cells (Ludueña et al., 1994; Koiso et al., 1994). The phytotoxic effect resulted in inhibited radicle and plumule growth in rice, wheat and maize, along with abnormal swelling of roots and germ in rice seedlings (Wang et al., 2017). Transcriptomic analysis reveals that the toxin is produced before false smut ball formation and may interfere with rice defense activation by downregulating *OsTGA2* and *OsLOX2*, which are involved in PR1 activation and jasmonic acid biosynthesis, respectively (Hu et al., 2020). Another major toxin produced by *U. virens* is polyketide ustilaginoidins belonging to bis-naphtho-γ-pyrones. Nearly 27 derivatives of ustilaginoidins have been identified, and the ustilaginoidin synthesis (*ugs*) gene cluster has at least 14 genes, among which *UvPKS1*, a polyketide synthase, is a central regulator of ustilaginoidin biosynthesis (Li et al., 2019). In addition to toxin biosynthesis, it is involved in vegetative growth, sporulation, tolerance to cell wall damage and pathogenicity. Increased susceptibility to sterol biosynthesis inhibitor fungicides, azoxystrobin, in the *UvPKS1* deleted mutants have been reported (Hou et al., 2026). The toxin also exhibited phytotoxicity activity by inhibiting rice radicle elongation, and cytotoxic activity against human cancer cells (Lu et al., 2015). Further, targeting the central regulator of toxin biosynthesis can open up a new avenue for disease control. Recently, the third group of polyketide toxins produced by the fungus, sorbicillinoids, was identified, which exhibited diverse biological activities, such as phytotoxicity, cytotoxicity, antifungal and antibacterial activities (Meng et al., 2019). The sorbicillinoids biosynthetic gene cluster, encoded by *UvSorA* and *UvSorB*, is responsible for the biosynthesis of the toxin. The toxin is involved in inducing cell cycle arrest and apoptosis in eukaryotic cells (Yao et al., 2015; Xie et al., 2021). Investigating the impact of secondary metabolites on rice-associated beneficial microbiota and their role in suppressing host defense responses represents an important direction for future research.

## 4 Molecular dialogue between rice and *U. virens*

The advent of whole-genome sequencing has accelerated the functional characterization of pathogenicity genes and virulence effectors (Zhang et al., 2014). The mitogen-activated protein kinase (MAPK) and Protein kinase A (PKA) signaling pathways play an important role in pathogenicity by transmitting signals from outside the cell to the nucleus, activating target genes through transcription factors, thereby promoting hyphal growth, differentiation, conidiation, and virulence. The MAPK and PKA signaling pathways and transcription factors involved in the production of effectors and pathogenicity have been explained in detail by Yu et al. (2023). In addition to effectors that directly interact with host targets, effectors involved in histone modification through histone deacetylation, methylation, and phosphorylation have been reported to mediate virulence (Yu et al., 2023).

### 4.1 Early infection: host entry and immune evasion

The pathogen establishes itself within the host by producing effector molecules that interfere with host recognition, reprogram hormonal pathways, hijack sugar transporters to facilitate nutrient acquisition, and suppress immune responses. To sustain a biotrophic relationship, the pathogen mimics fertilization processes, thereby keeping the ovaries viable and enabling continuous nutrient transfer from the host (Song et al., 2016). The lack of specialized nutrient-absorbing structures such as haustoria, along with its intercellular growth within floral tissues, suggests that the pathogen obtains nutrients from the extracellular apoplastic space (Tang et al., 2013). Whole-genome sequencing has revealed a limited repertoire of host-cell cell wall-degrading enzymes (CWDEs), alongside a diverse set of secreted effectors that manipulate host grain-filling processes to support colonization (Zhang et al., 2014). This constrained CWDE arsenal appears to have driven the evolution of a unique floral infection strategy.

**Figure 1:**
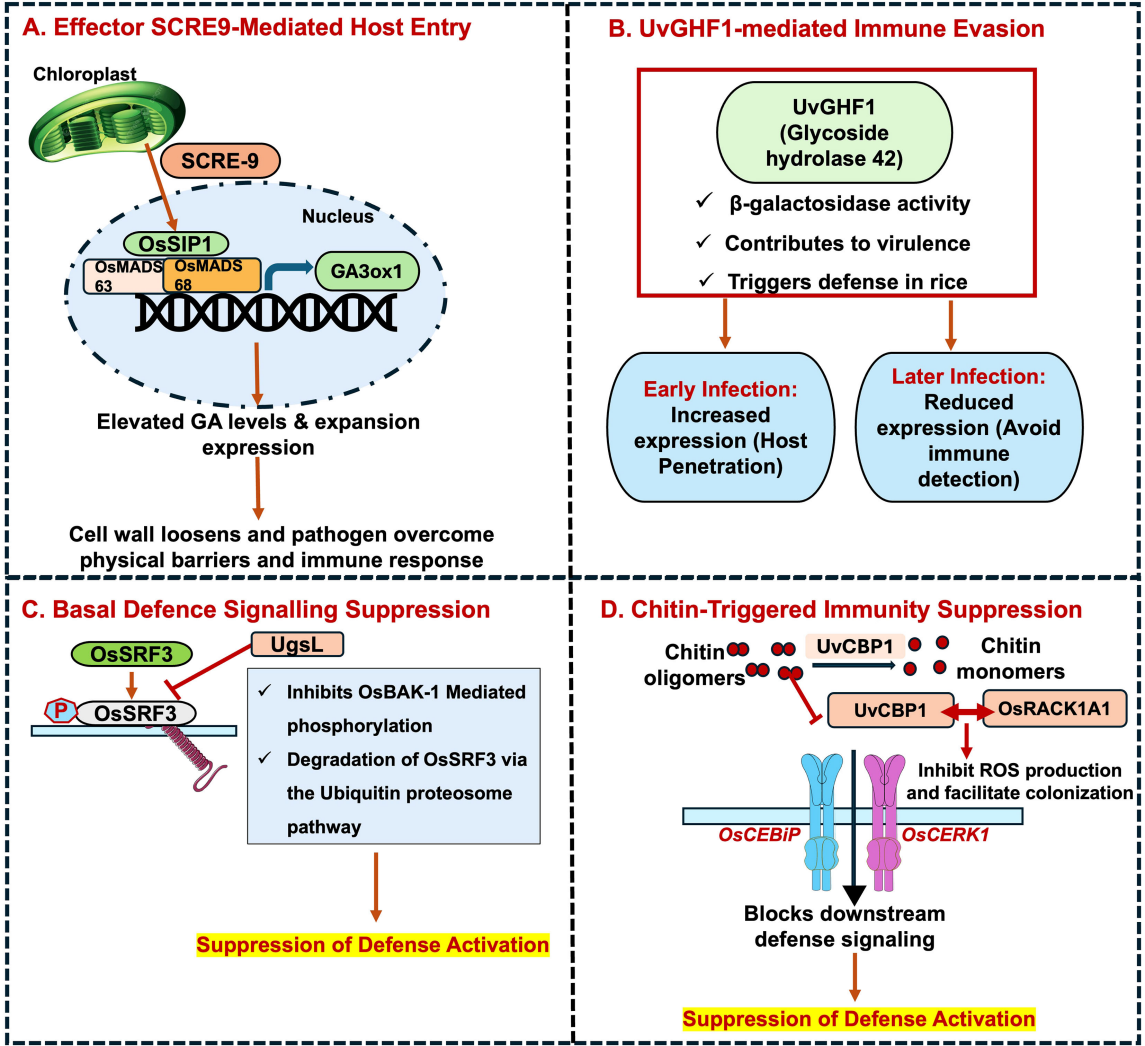
Effector-mediated infection and immune suppression by *Ustilaginoidea virens* in rice. **(A)** *UvSCRE9* promotes host entry by activating *GA3ox1* via *OsSIP1* and *OsMADS* transcription factors.; **(B)** *UvGHF1* exhibits stage-specific expression to aid penetration and later evade immune detection.; **(C)** *UgsL* suppresses basal defense by inhibiting *OsBAK1*-mediated signaling and promoting OsSRF3 degradation.; **(D)** *UvCBP1* interferes with *OsCEBiP-OsCERK1*-mediated chitin recognition, suppressing defense responses.

The secreted effector, *UvSCRE9*, induces the relocation of the chloroplast-localized *OsSIP1* protein to the nucleus. *OsSIP1* further recruits transcription factors *OsMADS63* and *OsMADS68*, which activate the gibberellic acid (GA) biosynthesis gene *GA3ox1* (**Figure 1A**). Elevated GA levels and expensing expression in florets lead to cell wall loosening, enabling the pathogen to overcome both physical barriers and immune responses (Yu et al., 2025). The virulence factor, glycoside hydrolase 42 (*UvGHF1*), which exhibits β-galactosidase activity, contributes to virulence and also triggers defense responses in rice (Song et al., 2021). Increased expression of *UvGHF1* during early infection reveals its role in host penetration, while reduced expression at later stages helps avoid immune detection (**Figure 1B**) (Zou et al., 2023).

The pathogen further evades host immunity by disrupting basal defense signaling. The ustilaginoidin synthetase effector *UgsL* inhibits OsBAK1-mediated phosphorylation of the receptor OsSRF3 and promotes its degradation via the ubiquitin-26S proteasome pathway, thereby suppressing defense activation (**Figure 1C**) (Duan et al., 2024a). Another cytoplasmic effector, *UvCBP1*, interacts with the scaffold protein *OsRACK1A* to inhibit reactive oxygen species (ROS) production, facilitating colonization (Li et al., 2022). Subsequent work identified *UvCBP1* (renamed *UvGH18.1*) as a chitinase that degrades immunogenic chitin oligomers into non-immunogenic monomers, effectively suppressing chitin-triggered immunity (**Figure 1D**) (Li et al., 2024). It also interacts with the chitin receptor *OsCEBiP* and its co-receptor *OsCERK1*, further blocking downstream defense signaling. Similarly, mannosyltransferases encoded by *UvALGs* contribute to immune suppression by sequestering chitin oligosaccharides, preventing their recognition and the activation of defense responses (Wang et al., 2025).

### 4.2 Modulation of rice immune signaling and response

To date, 421 putative effectors have been identified, although only a limited number have been functionally validated using reverse genetic approaches (Zhang et al., 2021). Several small cysteine-rich effectors (*OsSCREs*) have been reported to modulate plant immune responses (Zhang et al., 2014). The central rice signaling molecule *SnRK1a* activates PAMPs and positively regulates immunity by phosphorylating *XB24*, thereby enhancing its ATPase activity. However, the effector *UvSCRE1* competes with *SnRK1a* for binding to *XB24*, inhibiting ATPase activity and increasing host susceptibility, ultimately manipulating defense signaling pathways (Yang et al., 2022; Zhang et al., 2020). The nuclear effector *UvSCRE4* suppresses the activity of the auxin response factor *OsARF17*, which plays a key role in ROS burst and MAPK-mediated immune responses (Qiu et al., 2022). *Uv*S*CRE6* promotes pathogen colonization by dephosphorylating the negative immune regulator *OsMPK6* (Van Dingenen, 2022). Similarly, the nuclear-localized effector *UvSCRE7* competes with transcription factors *OsLBD11/12* for binding to the defense-related gene *OsCPS2*, preventing activation of immune responses (Jiang et al., 2026).

More recently, *UvSCRE2* has been shown to inhibit RNAi-mediated resistance in rice by binding to *OsRNS4*, an S-like ribonuclease, and blocking its interaction with host miRNAs that target the *UvSfk1* virulence gene in *U. virens* (Gao et al., 2026). A serine protease subtilase effector, *UvPr1a*, suppresses plant immunity by directly degrading the rice protein OsSGT1, which is involved in resistance pathways (Chen et al., 2022a). The secreted effector *UvSec117* inhibits jasmonic acid (JA)-mediated defense by preventing the interaction between *OsWRKY31* and the JA biosynthesis gene promoter *OsAOC* (Duan et al., 2024b). Additionally, UvSec117 interacts with the histone deacetylase *OsHDA701*, promoting hypoacetylation of histone H3K9 and further suppressing plant defense (Chen et al., 2022b). Another intracellular effector, *Uv1809*, targets the histone deacetylase *OsSRT2*, altering acetylation levels at *H4K5ac* and *H4K8ac* and negatively regulating defense responses (Chen et al., 2024). The lipid transfer protein *LTP113*, which is involved in pollen development and lipid-mediated immunity, is disrupted by the apoplastic effector *Sxp1* (secreted in xylem protein 1) (Xu et al., 2026).

### 4.3 Sugar transporter hijacking and nutrient acquisition

**Figure 2:**
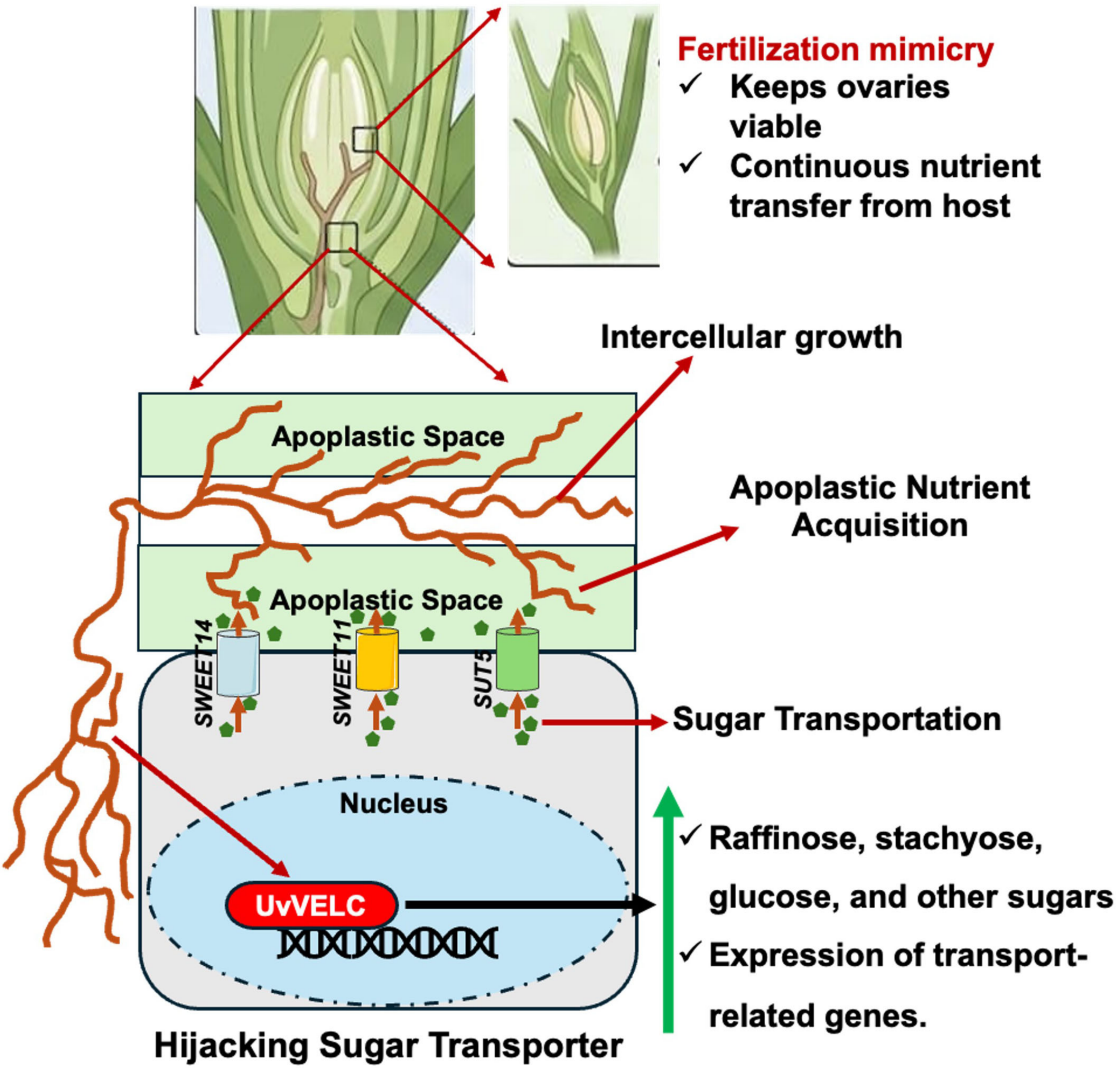
Putative nutrient hijacking by *Ustilaginoidea virens*. The pathogen putatively hijacks host sugar transporters (*OsSWEET11, OsSWEET14, OsSUT5*) to redirect nutrients into the apoplast, while potentially employing fertilization mimicry and *UvVELC*-mediated regulation to sustain infection and smut ball development.

Similar to the bacterial leaf blight pathogen, the false smut pathogen also recruits host sugar transporters to divert nutrients for its growth. It hijacks host sugar transporter genes such as *OsSWEET14, OsSWEET11, and OsSUT5*, facilitating the export of sugar molecules from the stamen into the apoplastic space to support *U. virens* growth during pathogenesis (**Figure 2**) (Fan et al., 2020). However, the virulence genes targeting sugar transporters are yet to be identified. Further, genes encoding raffinose synthase and galactinol synthase 2 were induced during infection, emphasizing that the pathogen utilizes raffinose or stachyose as a carbon source (Wang et al., 2016). Moreover, the *UvVELC* effector was identified, playing a key role in the utilization efficiency of raffinose, stachyose, glucose, and other sugars, as well as the expression of transport-related genes (**Figure 2**). This confirms that the false smut pathogen recruits host genes and effectors to transport nutrients to the apoplastic space (Yu et al., 2024). The conserved effector *UvHrip1* suppresses cell-mediated defense responses by inhibiting ROS production (Li et al., 2020). It also hijacks plant flowering time and grain development by interacting with the *OsHGW* gene, which is involved in heading date and grain weight signaling pathways. Further research may elucidate the mechanism underlying the fertilization mimicry phenomenon (Wei et al., 2020) (**Table 1**).

**Table 1:** Pathogenicity-related genes involved in the pathogenesis of *Ustilaginoidea virens* and their function.

Gene name	Family	Function	Reference
*UvSCRE9*	Small Secreted Cysteine-Rich Effector	Involved in gibberellic acid biosynthesis to loosen up the cell wall, enabling easy penetration	Yu et al., 2025
*UvGHF1*	Glycoside hydrolase	Exhibit β-galactosidase activity facilitating penetration	Zou et al., 2023
*UgsL*	Ustilaginoidin synthetase	Promotes degradation of the OsSRF3 receptor, suppressing defense activation	Duan et al., 2024a
*UvGH18.1*	Glycoside hydrolase/Chitinase	Degrades immunogenic chitin oligomers into non-immunogenic monomers, suppressing chitin-triggered immunity	Li et al., 2024
UvALGs	mannosyltransferases	Sequesters chitin oligosaccharides, preventing recognition and activation of defense responses	Wang et al., 2025
*UvSCRE1*	Small Secreted Cysteine-Rich Effector	Competes with SnRK1a for binding to XB24 and inhibits ATPase activity	Yang et al., 2022; Zhang et al., 2020
*UvSCRE4*	Small Secreted Cysteine-Rich Effector	Suppresses the activity of OsARF17, inhibiting ROS production and defense response	Qiu et al., 2022
*UvSCRE6*	Small Secreted Cysteine-Rich Effector	Activates the negative immune regulator OsMPK6	Van Dingenen, 2022
*UvSCRE7*	Small Secreted Cysteine-Rich Effector	Competes with transcription factors OsLBD11/12 for binding to the defense-related gene OsCPS2	Jiang et al., 2026
*UvSCRE2*	Small Secreted Cysteine-Rich Effector	Inhibit RNAi-mediated resistance	Gao et al., 2026
*UvPr1a*	Serine protease subtilize	Degrading the rice protein OsSGT1	Chen et al., 2022a
*UvSec117*	–	Modulates histone acetylation, suppressing defense Inhibits jasmonic acid (JA)-mediated defense	Chen et al., 2022b; Duan et al., 2024b
*Uv1809*	–	Alters histone acetylation	Chen et al., 2024
Sxp1	Secreted in xylem protein 1	Inhibit lipid-mediated immunity	Xu et al., 2026
UvPal1	–	Interacts with the cell division control protein 11, UvCdc11	Chen et al., 2020
*UvVELC*	Velvet family regulatory protein	Involved in the utilization of raffinose, stachyose, glucose and other sugars	Yu et al., 2024
*UvHrip1*		Inhibits ROS production, modulate heading date and grain weight signaling pathways	Li et al., 2020; Wei et al., 2020

## 5 Leveraging epidemiological factors for disease management

Early and precise detection of false smut enables growers to implement timely management practices and prevent financial losses, as the pathogen specifically targets floral organs during the booting stage under high humidity, rainfall, and moderate temperatures, with symptoms appearing only after panicle emergence when control becomes difficult. The use of epidemiological factors to develop forecasting models, along with in-field detection techniques, will help control the disease beforehand. Sathiyaseelan et al. (2025) identified temperature and precipitation during the booting stage as important climatic factors for the occurrence of disease. The daily temperature response (*f(T)* > 20) and daily precipitation (>5 mm) influenced disease occurrence (Sathiyaseelan et al., 2025). Another study conducted by Saha et al. (2020) identified spore concentration during the cropping season as one of the important factors, along with temperature and rainfall. In addition to forecasting models, spectral imaging can be used to detect the early occurrence of disease. Through UAV hyperspectral imaging, false smut disease can be monitored at the characteristic bands between 698–800 nm and 974–997 nm with reduced data noise (Wang et al., 2023). Near-infrared spectroscopy hyperspectral imaging (NIR-HIS) was used as a reliable technique to detect infected grain kernels with a detection accuracy of 89-91% (Wu et al., 2020). In the coming age of artificial intelligence, various machine learning algorithms can be trained to develop disease prediction, along with multispectral imaging to detect region-specific early disease occurrences. The in-field installed spore trapper, in combination with in-field isothermal amplification, provides the grower information about spore concentration and applies timely management practices to prevent losses. Loop-Mediated Isothermal Amplification (LAMP) assay could detect the pathogen at 100femtograms concentration, revealing the spatio-temporal distribution of the pathogen in an area (Arumugam Gopalakrishnan et al., 2024). Cultural practices, including field sanitation and removal of alternative weed hosts (*Panicum* spp., *Digitaria marginata*, *Echinochloa crus-galli*), along with agronomic adjustments, including avoiding overhead irrigation during booting, balanced nitrogen fertigation to prevent excessive vegetative growth, and strategic planting dates to avoid booting coinciding with peak monsoon conditions, can suppress the disease occurrence.

## 6 Towards sustainability

CRISPR-Cas genome editing offers a promising strategy to develop resistance against various plant diseases (Jaganathan et al., 2018). From the host perspective, CRISPR-Cas can be used to edit or knock out susceptibility (S) genes or negative regulators of immunity that facilitate fungal colonization, thereby restricting pathogen establishment and spread (Zaidi et al., 2018). For instance, disruption of TALE-binding elements of *OsSWEET11* and *OsSWEET14* using CRISPR/Cas9 in rice resulted in resistance to many *Xanthomonas oryzae* pv. *oryzae* (*Xoo*) strains (Xu et al., 2019). While editing promoter regions of *SWEET11*, *SWEET13*, and *SWEET14* genes targeted by the TALE effector of *Xoo* strains led to broad-spectrum resistance with agronomic performance (Oliva et al., 2019). Likewise, the host target, such as *OsMPK6*, which, when overexpressed, increases susceptibility to false smut, can be a potential target for CRISPR-based editing to manage the disease (Li et al., 2025). Similarly, CRISPR/Cas9-mediated overexpression of *OsSRF3*, which interacts with an effector *UgsL*, a ustilaginoidin synthetase responsible for *OsSRF3* degradation, conferred resistance in rice plants against the pathogen (Duan et al., 2024a). The overexpression of *OsRACK1A* in rice promoted ROS-mediated defense and conferred floral resistance without compromising yield, which is otherwise disturbed by *U. virens* secreted effector *UvCBP1* (Li et al., 2022). Further, modification of the *RBL1* gene resulted in the creation of a novel allele, RBL1Δ12, showing broad-spectrum resistance against false smut and bacterial blight (Sha et al., 2023). Even though direct targeting of fungal genomes within plant tissues remains challenging, few attempts were made previously (Zaidi et al., 2018). The efficient targeted gene knockout of the USTA gene (encoding ustiloxin) and the MAP kinase gene *UvSLT2* led to impaired fungal growth, conidiation, and stress responses in *UvSLT2* mutants, providing a foundation for developing innovative strategies to manage rice false smut (Liang et al., 2018). Similarly, deletion of *Uvpks1* not only abolished ustilaginoidin production but also altered secondary metabolism, reduced fungal growth, sporulation, stress tolerance, and pathogenicity (Hou et al., 2026). Recent advances in CRISPR-based editing emphasize its potential for developing broad-spectrum resistance against false smut in rice.

The phyllosphere microbiome (PM) is also being exploited and has been found to play a crucial role in suppressing rice false smut. PM combination of *Pantoea agglomerans*, *Acidovorax wautersii*, and *Bacillus pyrrocinia* resulted in a strong reduction in disease symptoms of false smut in rice (Wang et al., 2024). In addition, PM such as *Lactobacillus* spp. and *Aspergillus* spp. have been found to induce defense response, and a branched-chain amino acid (BCAA) leucine, remarkably repressed the *U. virens* infection by activating H_2_O_2_ production and quick cell death (Liu et al., 2023). Nevertheless, despite these promising biological approaches, their field-level consistency and scalability remain variable, necessitating continued reliance on chemical control strategies.

Even though in the last decade attempts to identify and introgress quantitative resistance loci have been made, complete resistance has not been found. Several QTLs have been identified from IR28, Lemont and RYT2668, such as *qFsr*10, *qFsr*12, *qFSR-6-7, qFSR-10-5, qFSR-10-2, qFSR-11-2*, *qRFSr-5.1, qRFSr-5.2* and *qRFSr9.1* in the majority of rice chromosomes (Xu et al., 2002; Zhou et al., 2014; Andargie et al., 2018; Neelam et al., 2022). Currently, control of false smut has largely relied on chemical fungicides. Several new-generation fungicides such as pydiflumetofen, propiconazole, tebuconazole, trifloxystrobin, azoxystrobin, and Difenoconazole have been documented to exhibit remarkable efficacy under field conditions (Thapa et al., 2026; Sharma et al., 2024; Kumar et al., 2020; Muniraju et al., 2017). However, the emergence of fungicide resistance in *U. virens* has become a major challenge. For instance, resistance to carbendazim has been reported to have developed in a number of strains of *U. virens* in China (Song et al., 2022). Fungicidal resistance against azoxystrobin, prochloraz and demethylation inhibitor (DMI) fungicides is attributed to overexpression of the *CYP51* gene (Fang et al., 2023; Pan et al., 2020). A recent study also advocated a high risk of resistance development in *U. virens* against albendazole (Li et al., 2026). The rapid emergence of resistance in *U. virens* poses a significant threat to their long-term sustainability. Therefore, integrating chemical control with alternative approaches such as biological control, resistance breeding, and genome editing for developing durable and sustainable management strategies for rice false smut is necessary.

## 7 Concluding statement

False smut of rice, caused by *U. virens*, has become a serious problem in rice-growing countries, yet its management remains ineffective and solely depends on the precise timing of using chemical fungicides. Despite advances in identifying effectors, virulence factors, and epidemiological drivers, there is no comprehensive understanding of the mechanisms linking molecular pathogenicity to field-level disease control, and durable host resistance remains absent. Future research should therefore focus on the functional characterization of key effectors and their host targets, particularly those involved in fertilization mimicry and nutrient reprogramming, alongside the identification of host susceptibility (S) genes for genome editing and resistance breeding. Integrating epidemiological models with real-time detection tools will be critical for predictive disease management. Additionally, targeting essential fungal genes, including those involved in toxin biosynthesis, and monitoring fungicide resistance will be crucial for long-term disease mitigation.
